# Comparison of Chemical and Mechanical Prophylaxis of Venous Thromboembolism in Non-surgical Mechanically Ventilated Patients

**DOI:** 10.7759/cureus.19548

**Published:** 2021-11-13

**Authors:** Fahad Ajmal, Mohammad Haroon, Umar Kaleem, Aisha Gul, Jawad Khan

**Affiliations:** 1 Critical Care Medicine, Bahria International Hospital, Rawalpindi, Rawalpindi, PAK; 2 Internal Medicine, Bahria International Hospital, Rawalpindi, Rawalpindi, PAK

**Keywords:** intensive care units, mortality, venous thromboembolic events, mechanical prophylaxis, chemical prophylaxis

## Abstract

To compare the efficacy of mechanical and chemical prophylaxis in non-surgically mechanically ventilated patients in terms of reduction in mortality and length of hospital stay.

A total of 200 patients admitted to intensive care units (ICUs) were recruited retrospectively. Half participants received mechanical prophylaxis and half received chemical prophylaxis. Patients with medical diseases with age 18 years or above, both genders, Pakistani nationals, receiving mechanical ventilation for more than 48 hours or receiving subcutaneous low molecular weight (LMW) heparin or subcutaneous unfractionated heparin were included. Cases who undergone surgery and were then admitted to ICU, those who received both mechanical and chemical therapies, and patients who received anticoagulant treatment before admission to ICU were excluded from the study. The patient’s age, gender, length of stay in ICU, and mortality were recorded in each group. Chi-square test was used to compare categorical data and Student t-test for continuous variables.

The mean age was 55.51±8.37 years. The males were 108(54%) and females were 92(46%). The mortality rate was higher in the mechanical prophylaxis group (49%) than chemical (31%) statistically significantly (P=0.014). Similarly, the length of hospital stay was also higher in the mechanical prophylaxis group (7.27±0.897 days) than chemical (6.67±1.045) statistically (P<0.001).

Chemical prophylaxis can reduce mortality and length of hospital stay more effectively than mechanical prophylaxis in ICUs admitted patients.

## Introduction

Venous thromboembolic events (VTEs) are among the most significant problems in medical patients, and they are common causes of morbidity and mortality [1,2]. VTEs include deep vein thrombosis and pulmonary embolism [3]. They are complications that occur in hospitalized patients, and they can be prevented but require substantial cost [4].

Two strategies can be used to reduce the risk of VTEs. One strategy is patient screening using accurate diagnostic tests. If an acute VTE is diagnosed early using screening, its progression can be prevented, thereby reducing its mortality and morbidity. Contrast venography is a diagnostic screening method; however, it is expensive, painful for patients, and limited in practicability outside clinical research [5]. Venous ultrasonography is a less invasive screening method with limited sensitivity, especially in asymptomatic patients. Its limited sensitivity in asymptomatic patients may be due to the presence of small non-occlusive thrombi [6]. The other strategy is implementing active measures for the prevention of VTEs [7]. These measures include prompt post-surgical embolization and active prophylaxis through mechanical or pharmacological methods [8-11].

Mechanical VTE prophylaxis is the use of gradual compression stockings and intermittent pneumatic compression devices to prevent blood stasis in the legs [12,13]. Mechanical VTE prophylaxis carries no risk of bleeding, but the devices used are uncomfortable for patients and can damage the skin of the lower extremities [14,15]. An alternative to mechanical VTE prophylaxis is chemical VTE prophylaxis. Low-dose anticoagulant therapy can reduce the incidence of VTE in up to 80% of cases, but it is associated with a slight risk of bleeding [16,17].

Gaspard et al. compared chemical and mechanical VTE prophylaxes in non-surgical patients on mechanical ventilation in the USA in 2010. There were 329 patients in the chemical prophylaxis group and 419 patients in the mechanical prophylaxis group. They reported mortality rates of 34.3% and 50.6% in the chemical and mechanical prophylaxis groups, respectively (P=0.005) [18].

Although different methods of VTE prophylaxis have been compared in various populations, to the best of our knowledge, no study comparing chemical and mechanical VTE prophylaxes in non-surgical mechanically ventilated patients in intensive care units (ICUs) in our population have been conducted. Thus, the aim of this study was to compare the efficacies of mechanical and chemical VTE prophylaxes in non-surgical mechanically ventilated patients in terms of mortality rate and length of hospital stay.

## Materials and methods

This retrospective comparative study included 200 patients admitted to the ICU at Bahria Town International Hospital, Rawalpindi from January 1, 2021, to August 30, 2021, and selected using a non-probability consecutive technique. Ethical approval was obtained from the hospital review committee. Before data collection, all participants received detailed explanations regarding the study, and verbal informed consent was obtained.

Of the 200 patients, 100 received mechanical VTE prophylaxis (i.e., compression stocking; group I) and the other 100 received chemical VTE prophylaxis (i.e., subcutaneous low molecular weight [LMW] heparin or subcutaneous unfractionated heparin; group II). Patients with medical diseases, of either sex, of Pakistani nationality, and aged 18 years and above who received mechanical ventilation for more than 48 hours and received subcutaneous LMW heparin or subcutaneous unfractionated heparin were included in this study. Patients who underwent surgery before ICU admission, patients who received both mechanical and chemical VTE prophylaxes, and patients who received anticoagulant therapy before ICU admission were excluded from this study.

Patient age, gender, length of ICU stay, and mortality rate were recorded. Length of ICU stay is expressed in days. The mortality rate was defined as the number of deaths due to VTE per total number of patients in each group.

Data were analyzed using SPSS version 22 (IBM Corp., Armonk, NY). Mean and standard deviation was computed for continuous variables (e.g., age, length of hospital stay), while frequency and percentage were calculated for categorical variables (e.g., gender, mortality) in each group. The level of significance for all analyses was P≤0.05.

## Results

The mean age of patients in this study was 55.51±8.37 years (range: 40-70 years). There were 108 men (54%) and 92 women (46%). There were no statistically significant differences in gender and age between the two groups (P=0.887 and P=0.966, respectively). The detailed statistics are shown in Table 1.

**Table 1:** Comparison of baseline parameters between the mechanical and chemical prophylaxis groups *Chi-square test **Independent t-test

Variables		Mechanical prophylaxis group (n=100)	Chemical prophylaxis group (n=100)	P-value
Gender	Male - n(%)	55 (55%)	53 (53%)	0.887^*^
	Female - n(%)	45 (45%)	47 (47%)	
Age (years) mean ± S.D.		55.49±8.43	55.54±8.34	0.966^**^

The mortality rate was significantly higher in the mechanical prophylaxis group than in the chemical prophylaxis group (49% versus 31%; P=0.014). Similarly, the length of hospital stay was significantly higher in the mechanical prophylaxis group than in the chemical prophylaxis group (7.27±0.897 days versus 6.67±1.045 days; P<0.001, Table 2).

**Table 2:** Comparison of mortality rate and length of hospital stay between the mechanical and chemical prophylaxis groups *Chi-square test **Independent t-test

Outcomes		Mechanical prophylaxis group (n=100)	Chemical prophylaxis group (n=100)	P-value
Mortality	Alive - n(%)	51 (51%)	69 (69%)	0.014^*^
	Deceased - n(%)	49 (49%)	31 (31%)	
Length of hospital stay (days) mean ± S.D.		7.27±0.897	6.67±1.045	<0.001^**^

## Discussion

The aim of this study was to compare the efficacies of mechanical and chemical prophylaxes in non-surgical mechanically ventilated patients. Our findings showed that mortality and length of hospital stay were higher in the mechanical prophylaxis group than in the chemical prophylaxis group.

Deep venous thrombosis and pulmonary embolism are two common types of VTE and are leading causes of mortality and morbidity in patients admitted to ICUs [19-21]. Three factors involved in the pathogenesis of VTE are blood stasis, endothelial injury, and hypercoagulability, which are known as Virchow’s triad of thrombosis [22-24]. Mechanical and chemical prophylaxes are two common methods of VTE prevention [12].

The purpose of mechanical prophylaxis in the form of compression stocking is to prevent blood stasis and reduce the risk of a thromboembolic event. Compression devices clear blood from the lower extremities and promote continuous blood flow [25,26]. Chemical prophylaxis involves the use of anticoagulants to prevent blood hypercoagulability and, consequently, VTE [27,28].

We conducted this study retrospectively using patient records available in the department; therefore, selection bias can be expected. We compared the age and gender of patients in both groups and found showed no significant differences, indicating that age and gender are not confounders in this study. These findings are comparable to those reported in an earlier study by Gaspard et al. [18].

Mortality rate and length of ICU stay of admitted patients are common parameters used to determine the efficacy of VTE prophylaxis [18,27]. In this study, chemical VTE prophylaxis was found to be more effective at reducing mortality and length of hospital stay than mechanical VTE prophylaxis. Gaspard et al. [18] compared chemical and mechanical VTE prophylaxes in non-surgical patients on mechanical ventilation in the USA in 2010. The chemical VTE prophylaxis group included 329 patients, while the mechanical VTE prophylaxis group included 419 patients. Their results showed that, in terms of mortality rate and length of hospital stay, chemical VTE prophylaxis is more effective than mechanical VTE prophylaxis. These results are consistent with our findings in this study.

We used subcutaneous LMW heparin or subcutaneous unfractionated heparin for chemical VTE prophylaxis. The efficacy of these anticoagulants is well documented, and their safety profile and bioavailability are excellent [22,29,30].

The main limitation of this study is its retrospective design. Further prospective randomized controlled trials on this topic are necessary to confirm the efficacy of the most appropriate VTE prophylaxis.

## Conclusions

Within the limitations of this study, it can be concluded that chemical prophylaxis reduces the mortality rate and length of hospital stay of patients admitted to ICUs more effectively than mechanical prophylaxis. Therefore, based on our results, anticoagulants should be preferred over compression stocking for patients admitted to ICUs. However, proper case selection and individual clinical judgment should always be taken into consideration.
